# Polyhydroxyalkanoate Production on Waste Water Treatment Plants: Process Scheme, Operating Conditions and Potential Analysis for German and European Municipal Waste Water Treatment Plants

**DOI:** 10.3390/bioengineering4020054

**Published:** 2017-06-06

**Authors:** Timo Pittmann, Heidrun Steinmetz

**Affiliations:** 1 TBF + Partner AG, Herrenberger Strasse 14, 71032 Boeblingen, Germany; 2 Department of Resource Efficient Wastewater Technology, University of Kaiserslautern, Paul-Ehrlich-Str. 14, 67663 Kaiserslautern, Germany; heidrun.steinmetz@bauing.uni-kl.de

**Keywords:** biopolymer, municipal sewage plant, PHA, primary sludge, VFA

## Abstract

This work describes the production of polyhydroxyalkanoates (PHA) as a side stream process on a municipal waste water treatment plant (WWTP) and a subsequent analysis of the production potential in Germany and the European Union (EU). Therefore, tests with different types of sludge from a WWTP were investigated regarding their volatile fatty acids (VFA) production-potential. Afterwards, primary sludge was used as substrate to test a series of operating conditions (temperature, pH, retention time (RT) and withdrawal (WD)) in order to find suitable settings for a high and stable VFA production. In a second step, various tests regarding a high PHA production and stable PHA composition to determine the influence of substrate concentration, temperature, pH and cycle time of an installed feast/famine-regime were conducted. Experiments with a semi-continuous reactor operation showed that a short RT of 4 days and a small WD of 25% at pH = 6 and around 30 °C is preferable for a high VFA production rate (PR) of 1913 mg_VFA_/(L×d) and a stable VFA composition. A high PHA production up to 28.4% of cell dry weight (CDW) was reached at lower substrate concentration, 20 °C, neutral pH-value and a 24 h cycle time. A final step a potential analysis, based on the results and detailed data from German waste water treatment plants, showed that the theoretically possible production of biopolymers in Germany amounts to more than 19% of the 2016 worldwide biopolymer production. In addition, a profound estimation regarding the EU showed that in theory about 120% of the worldwide biopolymer production (in 2016) could be produced on European waste water treatment plants.

## 1. Introduction

Common plastic is derived from petrochemicals based on the limited natural resource petroleum. Besides the exploitation of natural resources, the use of plastic is responsible for major waste problems, as common plastic is non- or poor biodegradable [1].

Biopolymers present a possible alternative to common plastics. If they are fully biodegradable [2,3] their use not only allows the preservation of limited resources, but also suits the idea of sustainability.

**Figure 1:**
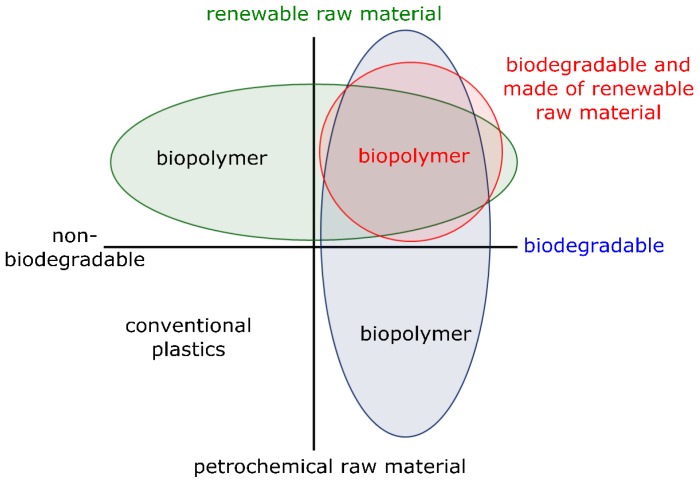
Definition for biopolymers, including the stringent definition on the upper right, modified after [4].

The term “biopolymer” or “bioplastic” is not yet uniformly defined. Common definitions of the term “biopolymer” also include biodegradable plastics from fossil fuels and non-biodegradable plastics from renewable resources as seen in Figure 1. To eliminate the problems accompanied by polymer production from crude oil a more stringent definition is introduced by the authors:

“Biopolymers are made from renewable resources and/or biodegradable waste materials (e.g., waste water, sewage sludge, organic waste) and are fully biodegradable by naturally occurring microorganisms.”

This definition ensures that polymers from fossil resources and non-biodegradable polymers, which cause at least one of the mentioned problems, are excluded and that the term biopolymer is just used for polymers, which allow the preservation of limited resources and also suit the idea of sustainability. This type of biopolymers is shown in the upper right of Figure 1.

Beside other polymers polyhydroxyalkanoates (PHA), which are biodegradable polyesters accumulated by bacteria under nutrient limited conditions [5] or under balanced growth, are a source for bioplastic production matching the above mentioned strict definition. More than 150 component parts of PHA have been identified so far [6]. The possibility for chemical modification of PHA provide a wide range of material properties and an even wider range of use [7,8]. However, so far the main raw material for the biopolymer production are starchy plants like maize [9], constituting the disadvantages of high land consumption, diminishing food resources as well as problems like leaching of nutrients, input of pesticide and soil erosion [10].

So far, municipal waste water treatment plants (WWTP) as alternative raw material and biomass source for the PHA production have not been widely investigated, although they offer the opportunity to compensate the disadvantages of the common PHA production using starchy plants.

**Figure 2:**
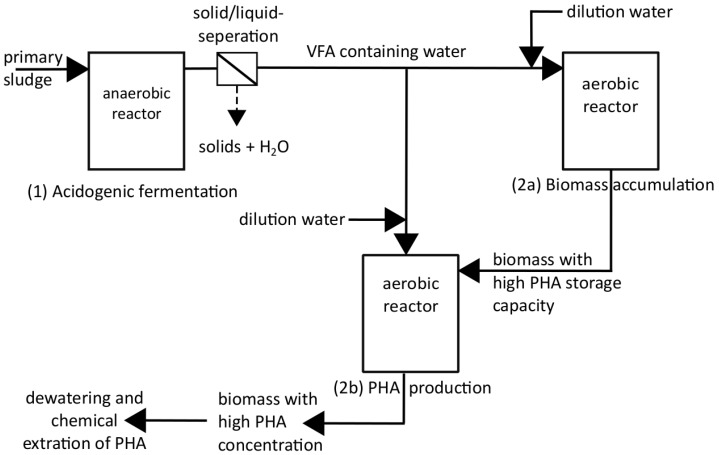
PHA production scheme.

PHA production in WWTP takes place in two steps, which composes the production of volatile fatty acids (VFA) in an anaerobic process and finally the PHA production in an aerobic process (see also Figure 2). In contrast to [11,12] the PHA production process described in this work is designed as a side stream process of a municipal WWTP and does not include the treatment of waste water. Therefore, the whole process can focus on polymer production only.

The possibility to use ice-cream waste water as alternative source material for the VFA production was shown by [13] while [14] investigated the effect of pH, sludge-retention time (RT) and acetate concentration on the PHA production from municipal waste water. Diverse authors [15,16,17,18] stated that there is a general possibility to produce PHA from activated sludge.

In many of the research projects on PHA production, synthetic waste water was used to gain knowledge about one part of the PHA production or the production’s operating conditions [19,20,21,22,23,24,25]. However, so far no research group has investigated the general possibility and all operating conditions of a biopolymer production using only material flows of a WWTP.

PHA production itself is based on a bacteria mixed culture selection from excess sludge via aerobic dynamic feeding. The installed feast/famine regime for enrichment of PHA producing bacteria is state of the art and tested by many authors [19,23,26,27]. The feast-phase is defined as a period of substrate availability and could be monitored via the reactors oxygen concentration. During the period of starvation (famine-phase) bacteria with the ability of polymer-storage gained a selection advantage as they are able to use the stored polymers as carbon and energy source.

The objective of this research project was to find the most suitable raw material and all operating conditions for the VFA and PHA production process using only material flows of a WWTP. At first the suitability of different raw materials of a municipal WWTP for VFA-production were investigated and afterwards the influence of operating conditions (temperature, pH, retention time (RT) and withdrawal (WD)) and reactor operation method. Another concern was, how the tested operating conditions or the diversity of the used material flows of a WWTP influence the VFA composition and the type of PHA produced. As there is a variation in the composition of the used material flows (different sludge) of a WWTP, it is of particular importance to observe their influence on VFA production and composition.

**Figure 3:**
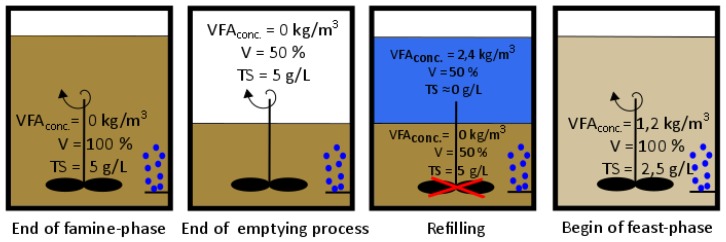
Emptying and refilling process during aerobic dynamic feeding (values shown are an example for investigated operation conditions), modified after [28].

Then the possibility to produce PHA out of the VFA containing substrate was tested using a feast/famine regime (as shown in Figure 3). Subsequently, the influence of operating conditions (temperature, pH, cycle time (CT) and substrate concentration) on PHA production were investigated.

The PHA potential on German WWTPs was calculated based on detailed data from operators of WWTPs [29] and the results of the PHA production experiments mentioned above. Finally, a profound estimation of the biopolymer potential of all WWTPs in the 28 member states of the European Union (EU) was made based on data provided by the EU [30] and the mentioned PHA production experiments.

## 2. Materials and Methods

### 2.1. VFA Production

#### 2.1.1. Conception of the Experiments

For the VFA production ((1) Acidogenic fermentation in Figure 2) anaerobic reactors of different sizes (4 L, 15 L) were operated as batch reactors or as semi-continuous reactors. While the batch operation is defined as a one-time substrate filling at the beginning of the experiment with no withdrawal and refill during the test, a semi-continuously operation method allows to introduce and withdraw substrate to or from the reactor. Semi-continuously is defined as one-time substrate filling at the beginning of every cycle, e.g., daily within a test duration of one month.

All tests were conducted without sedimentation or biomass recirculation. Therefore, the hydraulic retention time equals the sludge age and both will be referred hereinafter as RT.

The raw material is the most important base for gaining high PHA production rates, so that the selection of suitable raw material has the number one priority. The improvement of the VFA production’s operation conditions was examined afterwards with the most appropriate raw material found. A chronological test order was implemented as follows:

1. Selection of raw material
2. Investigation of the most suitable pH-level
3. Evaluation of a retention time (RT) range
4. Selection of a suitable combination of RT and withdrawal (WD)

#### 2.1.2. Selection of Raw Material

For raw material selection continuously stirred batch reactors with a volume of 4 L were used. Four different types of sludge, namely primary sludge (average total solid TS_a_ = 43 g/L), excess sludge (TS_a_ = 10 g/L), a one to one mixture of primary-and digested sludge (TS_a_ = 37.5 g/L) and a one to one mixture of excess- and digested sludge (TS_a_ = 21 g/L) from a municipal WWTP were treated under anaerobic conditions. Thereby the digested sludge from the WWTP’s digester was only used as inoculum for the anaerobic process in order to find out, if it could accelerate the process. All types of sludge were investigated under four different conditions: pH controlled at pH = 6, without pH-control and each at around 20 °C or around 30 °C reactor temperature. In summary 16 different tests were performed. The reactors were filled at the beginning of the experiments and samples of 50 mL were retrieved every day to determine the VFA concentration and composition. To achieve the selected temperature, the reactors were situated in temperature-controlled rooms. For pH-controlled tests, the pH-value was measured by a mobile pH meter (WTW pH340i) and adjusted with NaOH by hand twice a day. The test duration for all experiments was 18 days to 20 days. A sample of all tested types of sludge was taken before and after the tests to determine the chemical oxygen demand (COD), the total Kjeldahl nitrogen(TKN) and total P.

#### 2.1.3. Evaluation of Operating Conditions

As the influence of fermentation temperature was already observed during the selection of the raw material, three additional operating conditions (pH, RT, WD) were investigated in continuous stirred tank reactors (CSTR) with a volume of 15 L. For all experiments primary sludge from a municipal WWTP was used as raw material and a sample was retrieved prior to the experiments to determine COD, TKN and total P. All reactors were placed in a temperature-controlled room at around 30 °C. pH was monitored at all times via a Metrom Profitrode pH probe and automatically adjusted with NaOH during all tests.

For the batch tests (pH, pre-RT) all reactors were filled with primary sludge at the beginning and samples of 100 mL were retrieved every day to determine the VFA concentration and composition. The batch test period was 18 days to 20 days long. Former studies show a high VFA production in a pH-range between 5 [11] and 9 [31] or 11 [32]. In consequence a range of pH-levels (pH = 6, 6.5, 7, 8, 10) were tested.

For the semi-continuously operated tests all reactors were filled with primary sludge at the beginning and operated under pH-controlled conditions at pH = 6. After a starting phase of 10 days, to accumulate VFAs, the semi-continuous operation phase began, for which a certain amount of the sludge in the reactor was exchanged. Samples of 100 mL were retrieved to analyse the VFA concentration and composition for about 40 days with a RT of 4 days, 6 days and 8 days, each with 25% and 50% WD. Additionally, a 75% WD was performed with a RT of 4 days. A RT of 2 days was also tested with a WD of 50%. RT and WD are related factors, e.g., a RT of 4 days was used when 25% of the sludge was exchanged every day, 50% every second day or 75% every third day.

#### 2.1.4. Analytical Procedures

COD, TKN, total P and total solids (TS) were determined according to standard methods.

The concentration and composition of volatile fatty acids, namely formate (Fo), acetate (Ac), propionate (Pro) and butyrate (Bu), were detected by high performance liquid chromatography (HPLC). Therefore, the sample was acidified to pH = 2 and filtered through 0.45 µm membrane filter. Afterwards HPLC detection was performed using a HP1100 chromatographer equipped with an UV detector and a Varian Metacarb 87H column. Sulphuric acid (0.05 M) was used as eluent at a flow rate of 0.6 mL/min. The detection wavelength was 210 nm. Volatile fatty acid’s concentration was calibrated using 4 nmol to 4000 nmol standards.

As the results of formic acid detection was below detection point for all except one test, formic acid is not shown in the VFA composition.

#### 2.1.5. Conversion of Units

For a better comparability all results regarding VFA concentrations, COD, TKN and total P were converted into mg/L.

The concentration of VFA in terms of mg/L is defined as:

VFA = Ac + Pro + Bu
(1)

The degree of acidification (DA) was calculated according to [33] as shown in Equation (2). As VFA results are given in mg_VFA_/L they have to be converted into COD units as shown in Equation (3)

DA = VFA/COD_S_ in (mg_COD_/L)/(mg_COD_/L) (2)

with COD_S_ = COD of Substrate at the start.

COD_VFA_ = (conc. VFA_i_/molar mass VFA_i_) oxygen demand (3)

with i = Ac, Pro, Bu.

For a better comparability regarding the different RT and WD, the average of the VFA concentration during the test period was calculated. In a second step, the average VFA concentration was used to calculate the average VFA production rate (PR_VFA_). This step also eliminates the reactors size and can hence be considered as average VFA production rate per day and litre, which will be referred to production rate (PR) hereinafter (Equation (4)).

PR_VFA_ = av. VFA conc./RT in mg_VFA_/(L×day) (4)

This calculation helps to compare results from different reactor sizes and retention times.

### 2.2. PHA Production

#### 2.2.1. Experimental Set-Up

The overall process describing the production of biopolymers from municipal waste water is displayed in Figure 2. For PHA production only “(2a): Biomass accumulation” and “(2b): PHA production” is considered. The first step, the volatile fatty acids (VFA) production, was already discussed in Chapter 2.1. The substrate produced in step 1 under anaerobic fermentation process at 30 °C, pH = 6, RT = 4 days and a withdrawal of 25% was frozen at −18 °C and defrosted about 24 h prior to its use as input material for phase 2 PHA production.

During all PHA production tests continuous stirred tank reactor (CSTR) with a volume of 15 L were used. All reactors were equipped with a pH- and oxygen-probe. If necessary, pH-value was adjusted via a dosing pump. pH levels were controlled by the pH probe and adjusted with NaOH or H_2_SO_4_. The reactor temperature was controlled via the pH-probe and manually adjusted using a heating bath (Haake DC30) and a heat exchanger, installed in the reactor. To maintain aerobic conditions an aerator was installed in the reactor. All reactors were operated in batch mode. The batch operation is defined as a one-time substrate filling at the beginning of the aerobic dynamic feeding-cycle with no withdrawal and refill during the cycle. There was no sedimentation or biomass recirculation in all tests. Therefore, the hydraulic retention time equals the sludge age and both will be referred hereinafter as RT. At the end of every cycle 7.5 L were withdrawn from reactor 2a and filled into Reactor 2b. Hence a RT of 2 days was implemented. Afterwards both reactors were filled with 7.5 L of substrate and fresh water to achieve the working volume of 15 L per reactor. Reactor 2b was emptied at the end of the feast-phase, and samples were taken to measure the PHA concentration and composition.

Both reactors were operated under similar conditions, just differing concerning nutrient availability. As the VFA enriched substrate showed nutrient limited conditions, with a Carbon:Nitrogen:Phosphorus (C:N:P)-ratio in a range of 100:2:0.5 to 100:3:0.8 [34] CH_4_N_2_O and KH_2_PO_4_ were added to Reactor 2a to create optimal conditions for the bacteria growth (C:N:P = 100:5:1) and selection process. Reactor 2b, however, was operated under the named nutrient limited conditions to reach a higher PHA concentration.

Samples to determine the PHA concentration were taken at the end of the feast-phase. The total solid (TS) concentration was measured at the end of each cycle and was about 5 ± 0.5 g/L in both reactors. This was done to figure out if the selection process in Reactor 2a was working correctly or if the biomass concentration was decreasing e.g., for lack of nutrients. As the biomass concentration was stable throughout all experiments no further tests regarding cell growth were conducted.

Experiments regarding the substrate concentration and temperature selection had the top priority. Afterwards the optimisation of all other operation conditions concerning the PHA production was examined with the most appropriate reactor temperature and substrate concentration found. A chronological test order was implemented as follows:

1. Selection of a suitable substrate concentration
2. Investigation of the reactor temperature
3. Evaluation of a suitable pH-level
4. Selection of a suitable cycle time

#### 2.2.2. Investigation Concerning the Best Substrate Concentration

As there is a big variation in substrate concentration in literature, different substrate concentration of 1200 mg_VFA_/L and 2000 mg_VFA_/L were tested at 20 °C or 30 °C, pH = 7 or without pH control and a CT = 24 h. The named concentrations were chosen to avoid possible problems triggered by substrate inhibition.

#### 2.2.3. Investigation Concerning the Best Temperature

The temperatures of material flows from a municipal WWTP are about 15 °C to 20 °C in temperate climates and might exceed 30 °C in hot climates or after mesophilic acidification. So tests with 15 °C, 20 °C and 30 °C were performed with a substrate concentration of 1200 mg_VFA_/L, pH = 7 and a CT of 24 h or 48 h to find the best reactor temperature regarding PHA production. To avoid a substrate induced influence all tests were conducted with the same substrate batch.

#### 2.2.4. Investigation Concerning the Best pH Level

As the best pH level to produce PHA depends on the used substrate, various pH levels (6, 7, 8, 9, without pH control) were tested in this study with a substrate concentration of 1200 mg_VFA_/L, a temperature of 20 °C and a CT = 24 h.

#### 2.2.5. Investigation Concerning the Best Cycle Time

To find the most suitable cycle time (CT) for the bacteria selection process, experiments with a substrate concentration of 1200 mg_VFA_/L were conducted at a temperature of 20 °C and at pH = 7 or 8. As the feast/famine ratio is more important than the overall CT a constant substrate concentration should ensure that the feast phases of the cycles were constant and the variation in CT resulted in a different famine phase, only. [25] stated that the feast-phase should not last longer than 20% of the overall CT to create a selection pressure on non-PHA accumulating bacteria. Therefore, CTs of 24 h, 48 h and 72 h were tested.

#### 2.2.6. Analytical Procedures

COD, TKN, total P and total solids (TS) were determined according to standard methods.

The concentration and composition of polyhydroxyalkanoates (PHA), namely polyhydroxybutyrate (PHB) and polyhydroxyvalerate (PHV), were detected by gas chromatography, according to [35] with some variations. Therefore, the biomass was separated via a centrifuge at 10,000 rpm for 20 min and dried at 105 °C. Afterwards the sample was pulverised with a ball mill and about 100 mg were digested and analysed. Detection was performed using a Perkin Elmer Autosystem XL chromatographer and a VF5ms 30 m × 0.25 column. Helium was used as carrier gas. PHA concentration was calibrated using 4 mL standards. The concentration and composition of volatile fatty acids, namely formate (Fo), acetate (Ac), propionate (Pro) and butyrate (Bu), were detected as described in Chapter 2.1.3.

#### 2.2.7. Calculation of Parameters

For better comparability, all results regarding VFA concentrations, COD, TKN and total P were converted into mg/L. The concentration of PHA in terms of % cell dry weight (CDW) is defined as:

PHA = PHB + PHV
(5)

### 2.3. Potential Analysis

#### Calculations

Based on the PHA production results described in Chapter 3, a potential analysis was performed. The aim of the analysis was to determine the potential of biopolymer production (based on renewal resources and biodegradable, see definition in Chapter 1) on German and European waste water treatment plants (WWTP) by using sewage sludge as a substrate. All input data used for the calculations can be found in Table 1.

A plausibility analyses was performed to cross-check the most important input data like the amount of primary sludge (PS) per population equivalent (PE).

As detailed data about waste water and sewage sludge production are available in Germany, the first step of the potential analysis was calculated using these data together with results presented in Chapter 3. In a second calculation step, data provided by the European Union (EU) were used to create an in-depth estimation of the biopolymer potential considering all 28 member states.

## 3. Results and Discussion

### 3.1. VFA Production

#### 3.1.1. Potential Analysis

Table 2 displays the results of the performed investigations ordered by degree of acidification. Primary sludge performed best under three out of four conditions and yielded by far the best degree of acidification (DA) with 31% at 30 °C under pH-controlled conditions [34]. The second best carbon source, a one to one mixture of primary and digested sludge at 20 °C under pH-uncontrolled conditions, achieved only a DA of 14%. In five out of eight experiments pH-uncontrolled conditions resulted in a higher DA. Therefore, a fermentation without pH control should be considered for all fermentation raw materials. However, primary sludge yielded better DAs with pH control at both investigated temperatures. At 30 °C the DA of primary sludge was twice as high than without pH control.

Table 2 also shows the composition of the VFA. The results varied strongly between 24/76/7 (%Ac/%Pro/%Bu) and 100/0/0 depending on the used raw material. Primary sludge produced none butyric acid and acetic and propionic acid in nearly two equal sections. Excess sludge on the other hand produced up to 21% butyric acid, while the one to one mixture of primary and digested sludge produced the most acetic acid (up to 84%) of all tested raw materials. The results show that the raw material has a major influence on the VFA composition.

As the use of primary sludge resulted in highest DA and showed only small variations in VFA composition under the tested conditions, it was chosen as raw material and used in all further tests.

Beside the ability to produce VFAs, primary sludge has other advantages as raw material for the PHA production. As primary sludge is a mixture of organic material, water and fermenting microorganisms no longsome biological adaptation-phase or biomass recirculation for the fermentation process was necessary. During all experiments primary sludge showed nutrient limited conditions, as described in Chapter 2.2.1. This is of particular significance given that nutrient limited conditions are essential for the later PHA production [19,37].

#### 3.1.2. Temperature

The aim of the investigations at two temperature levels was to ascertain if the VFA production at ambient temperature (20 °C) can reach the same VFA production compared with heating the sludge.

In six out of eight tested combinations a temperature increase from 20 to 30 °C caused a higher VFA production as shown in Table 2. The experiment confirmed the results of [32], who stated that the VFA concentration increases with higher fermentation temperature. Using primary sludge as raw material (under pH-controlled conditions) the temperature change from 20 to 30 °C caused a DA increase from 14% to 31%.

The general assumption that the acidification rate is higher at 30 °C than at 20 °C could not be confirmed. Only three out of eight tested combinations reached their VFA maximum at 30 °C in a shorter span of time than at 20 °C. Four out of them even reached their VFA maximum at 20 °C in a shorter span of time than at 30 °C. The results can be seen in Table 2. Primary sludge under pH-controlled conditions obtained its VFA maximum after 7 days at 20 °C and after 9 days at 30 °C. Nevertheless, the fact that the DA of primary sludge under pH-controlled conditions at 30 °C was twice as high as the DA at 20 °C is all the more important as the VFA production at 30 °C lasted only about 30% longer.

The variation of temperature has a wide range of influence on the VFA composition, depending on the used substrate. As primary sludge was already chosen as substrate for the optimisation tests, only its VFA composition was of interest for further tests. However, in the case of primary sludge the temperature change investigated resulted only in marginal changes in the VFA composition.

Consequently, a temperature around 30 °C for the further experiments was considered as reasonable.

#### 3.1.3. pH

**Figure 4:**
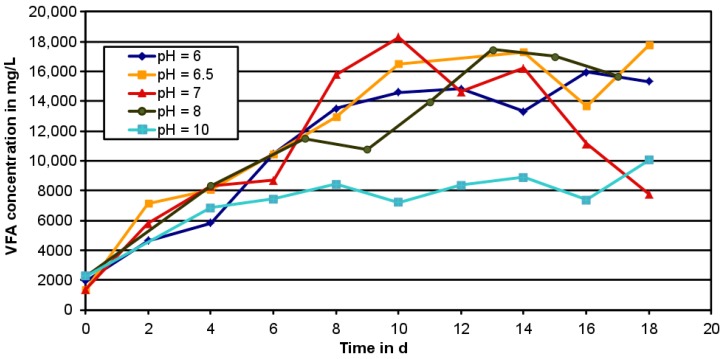
VFA concentrations during tests at different pH levels.

As illustrated in Figure 4, no big difference in the maximum VFA concentration between pH = 6 and pH = 8 was observed. A pH value of 7 yielded the highest result with 18,286 mg_VFA_/L after a RT of 10 days. The fermentation at pH = 10 reached significantly worse results with a maximum of 10,050 mg_VFA_/L only at 18 days retention time. This is in contrast to the results of [31] showing the best result at pH = 9 with excess sludge and food waste as source material and [32] yielding the highest result at pH = 11 with excess sludge as raw material.

Although pH = 7 yielded the best result, methane production turned out to be an issue at this pH-value. After about 15 days the acetate concentration was falling rapidly and the overall VFA concentration after 18 days was less than 44% of the maximum (Figure 4). During this period more than 15 Vol.% methane was detected in the reactor via gas measurement. To prevent methanogenic conditions a pH-level of 6 has to be kept [38]. Consequently, further investigations were performed under pH = 6, although it produced about 12% less VFA within the batch experiments.

The variation of pH-level showed a strong influence on the VFA composition. Changing the pH from 6 to 7 within the batch experiments caused a constant decrease in the acetic acid ratio as shown in Table 3. In the same tests, the propionic acid ratio increased, while the butyric acid ratio decreased to zero. At pH = 8 conditions a reverse trend was observed. With 60% the maximum acetic acid ratio as well as the minimum propionic acid ratio (37%) was detected, while butyric acid was produced in small amounts (3%). In comparison to pH = 8, a reduction of acetic acid and propionic acid production was detected at the highest tested pH-level (pH = 10), while the butyric acid ratio increased to the highest level (8%) observed. In contrast to the other pH-values tested, formic acid was produced at pH = 10 with a ratio of 16.

#### 3.1.4. RT and WD

To get an idea about how much adapted bacteria are needed in the reactor to produce the most VFAs a wide range of RT and WD was tested. RT = 2 days and WD = 50% yielded poor results (VFA_max_ < 2000 mg_VFA_/L) and after 10 days of semi-continuous operation the test was shut down. Therefore, these results are not shown. Further results ranged in a broad band between 5000 mg_VFA_/L and 10,000 mg_VFA_/L.

Obviously, the VFA production with short RTs and small WDs fluctuated less than using long RTs and large WDs, what can be explained by the changing composition of the introduced primary sludge. These changes of the used primary sludge are mostly due to weather events. A rainfall after a period of dry weather can transport a huge amount of organic matter to the WWTP. Daily changes in the waste water’s composition or different contents during the week could be another reason for changing the primary sludge’s composition. Smaller WD stabilises the fermentation process because only little material is turned over and the reactor is less sensitive to heterogeneous primary sludge input.

**Figure 5:**
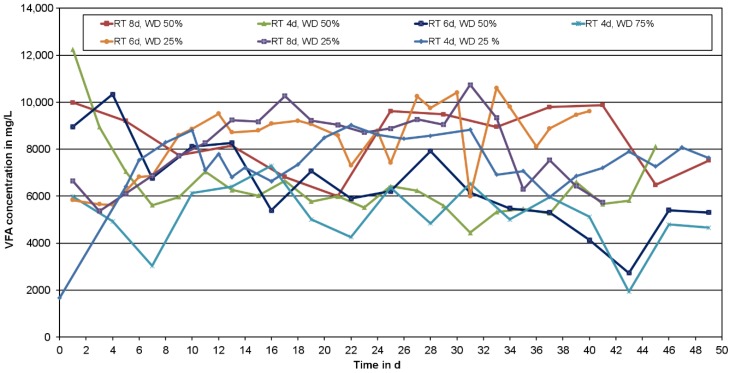
VFA concentration in dependence of RT and WD.

Figure 5 shows the average VFA concentration over a period of 40 days for all investigated combinations. Both, RT and WD influenced the VFA production. With higher WD the VFA concentration was decreasing at all tested RTs. The highest overall VFA concentration was reached at a RT of 6 days with a WD of 25%. Longer and shorter RTs (with a WD of 25%, too) resulted in lower VFA concentrations.

In order to have a high PHA-production in the second stage the VFA production rate (PR) is more important than the VFA concentration, which could, if it exceeds a certain value, lead to a substrate inhibition [23]. Therefore, the PR was calculated on Equation (4). A RT = 4 days and WD = 25% yielded the top production rate with PR = 1913 mg_VFA_/(L×d) at a VFA concentration of 7653 mg_VFA_/L on average [4].

The variation of RT influenced the VFA composition only slightly as shown in Table 3. Acetic acid and propionic acid were produced in a similar range, while butyric acid was always the smallest part. Nevertheless, the variation of WD had an effect. With a WD of 25% the fraction of propionic acid was about 20% smaller throughout all tests, compared to a WD of 50% and 75%, while the butyric acid ratio was nearly twice as much. The acetic acid ratio with a WD of 25% was slightly higher than for any other WDs.

**Figure 6:**
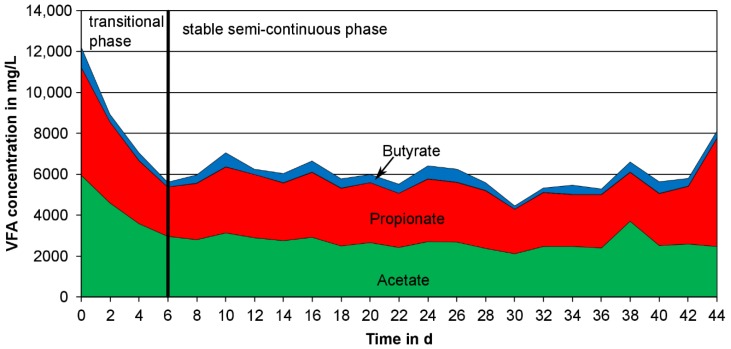
Development of the VFA concentration and composition at RT = 4 days, WD = 50%.

As a stable VFA composition is necessary for high quality PHA production the possible fluctuation of the VFA composition during the whole test period is important. Figure 6 is exemplary for all semi-continuously operated tests and shows the concentrations of acetate, propionate and butyrate at RT = 4 days, WD = 50% during the whole test period of 44 days. After a starting phase of 10 days (not shown in the figure) the semi-continuous operation began. Due to the change in the operation method (from batch to semi-continuously) a transition phase with a decrease in VFA concentration was observed for the first six days of semi-continuous operation. Fluctuations in the VFA concentration between day six and day 44 were due to the changing concentration and composition of the introduced primary sludge. Although a fluctuation in VFA concentration after the fermentation step was observed, only small changes in the VFA composition were detected. Thus, it was possible to show that the variability of the raw material primary sludge did not affect the VFA composition significantly.

### 3.2. PHA Production

The acidified primary sludge from phase 1 was used for the production of PHA in step two where the influence of different operating conditions was investigated.

#### 3.2.1. Substrate Concentration

The results of the conducted experiments are displayed in Table 4. It appears that higher PHA concentrations were gained at the lower substrate concentration tested. The maximum PHA concentration of 25.9% based on cell dry weight (CDW) was reached at a pH of 7 and a reactor temperature of 20 °C. With a VFA concentration of 2000 mg/L and a reactor temperature of 20 °C the highest PHA concentration achieved was 4.8% CDW, only and therefore much less than at a substrate concentration of 1200 mg/L at the same temperature. This confirms the observations of [8,23,39] that an increasing substrate concentration could result in a substrate inhibition.

Furthermore, the experiment at the higher temperature and the lower substrate concentration (30 °C, 1200 mg/L) leads to a significant lower PHA production than at 20 °C and 1200 mg/L. This indicates that a reactor temperature of 20 °C may be preferable for a primary sludge based PHA production (see also Chapter 3.2.2).

PHA composition did not show any dependence regarding to substrate concentration. During all tests higher proportions of PHB than PHV were produced. About twice as much PHB (than PHV) was produced at all experiments at a substrate concentration of 2000 mg/L and at 30 °C with 1200 mg/L, while a nearly equal proportion of both PHAs was reached at 1200 mg/L and 20 °C. 

#### 3.2.2. Temperature

**Figure 7:**
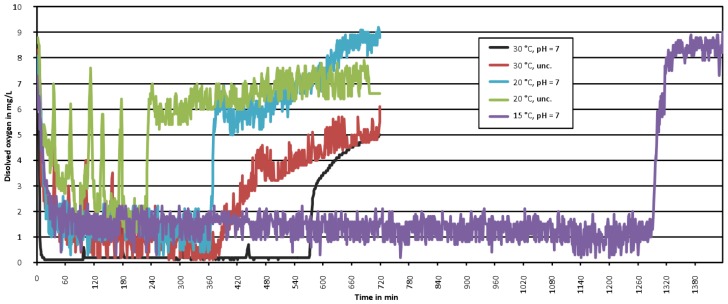
Reactor temperature influence on the feast/famine phase length.

Table 5 displays the results of the PHA production at different temperatures. As the bacteria metabolism is slower at lower temperatures a very long feast-phase was observed at 15 °C. To ensure a sufficiently long famine-phase the cycle time of 24 h was doubled at all test with a reactor temperature of 15 °C. Figure 7 shows the length of the feast-phase based on the reactors oxygen concentrations. The very long feast-phase at 15 °C (around 22 h) is clearly visible. All other feast-phases at 20 °C or 30 °C were significantly shorter and not longer than 600 min. A somewhat surprising fact was that a faster bacteria metabolism at 30 °C did not result in a shorter feast-phase than at 20 °C. These observations are in contrast to [40], who stated that shorter fest-phases are observed at higher temperatures. At the same time, they confirm the results of [41], who gained the highest PHA concentration at reactor temperatures around 20 °C. This effect may origin in the fact, that the used sewage sludge was already adopted to temperature around 20 °C.

There is no influence on PHA composition due to temperature changes. The results of Chapter 3.2.1 that the PHB/PHV ratio is about 2/1 at lower PHA concentrations produced, was confirmed (Table 5 at 15 °C and 30 °C), while both proportion are more or less equal at a higher PHA production (Table 5 at 20 °C).

As listed in Table 5 conditions of 20 °C yielded the highest PHA concentrations with 13.2% CDW and 25.9% CDW. At a reactor temperature of 15 °C or 30 °C a maximum PHA concentration of less than 4.5% CDW was reached, only. In consequence further experiments were conducted at 20 °C to achieve the best possible PHA concentration [42].

#### 3.2.3. pH

Table 6 illustrates big differences in the maximum PHA concentration between the tested pH levels. All tests were operated in batch-mode with a cycle time of one day. The substrate’s pH was adjusted before adding it into the reactor. An exception was the pH uncontrolled test. This experiment should clarify if a high PHA production is possible at fluctuating pH value. During the whole cycle the pH varied between 7.3 at the beginning of the feast-phase and 9.3 at the end of the famine-phase, with an average pH around 9.

The experiments with controlled pH values showed highly varying results. While there was no detectable PHA production at pH = 6, more than 25% CDW was produced between pH 7 and 8, with a maximum PHA production of 28.4% CDW at pH = 8. Then again, at the highest pH of 9 a low PHA production of 4.4% was reached only. The experiment without pH control produced around half as much PHA as the tests at pH 7 or 8. Nevertheless the results of the uncontrolled test were far better than at the same pH-level of 9 during the controlled test. Still a pH controlled PHA production is preferable, as the maximum PHA production was obtained at pH controlled operation [42].

pH changes did not influence the PHA composition. As described in Section 3.2.2 and Section 3.2.3 the PHB/PHV ratio is 2/1 at low PHA concentrations and nearly 1/1 at a higher PHA production.

#### 3.2.4. Cycle Time

When using a PHA production based on a bacteria mixed culture a feast/famine regime is crucial. This is the only way for PHA producing bacteria to gain a significant selection advantage over other microorganisms.

The produced amount of PHA in dependence of the length of the feast/famine-phase is displayed in Table 7. A correlation between the cycle time (CT) and the length of the feast-phase was observed, showing that a longer CT leads to a shorter feast-phase. This could be due to a longer and therefore harder famine-phase leading to a long period of starvation. It seems that after a bigger starvation, the bacteria’s substrate uptake is higher and faster than at shorter cycle times. [39] highlights the cycle time must be such that the complete PHA is metabolised at the end of the famine-phase. This guideline was confirmed by testing samples, taken at the end of each famine-phase. No PHA was detected and therefore the required operating condition was kept during all tests. Thus, a cycle time of 24 h is sufficient.

A former study concluded that the proportion of the feast-phase should not exceed 20% of the CT, because a longer famine phase could lead to a lower selection pressure on non-PHA accumulating bacteria [26]. Table 7 shows a feast-phase proportion of 36% at a cycle time of 24 h and therefore nearly twice as high as suggested by [26]. Regardless, at this cycle time the highest PHA concentration (28.4% CDW) was reached. A feast-phase proportion of 17% at a CT of 48 h led to a PHA production of 18.3% CDW, while at the longest tested CT of 72 h a PHA production of 21.4% CDW was observed with a feast-phase proportion of 10%. This indicates that a fixed feast-phase proportion is unnecessary and the demanded operation condition by [39] is preferable. However, more experiments will be necessary to confirm these results.

An influence of the cycle time length on the PHA composition is shown in Table 7, also. While all other experiments reached a higher PHB than PHV proportion a cycle time of 48 h or longer led to a higher PHV production. This could be due to the fact that the PHA accumulation bacteria produced PHB at first while the PHV production started later. As no samples were taken at low oxygen levels during the feast-phase and no uptake rate was measured for the VFAs (acetate, butyrate, valerate) during the experiments this presumption could not be confirmed.

The preferable operating conditions for the VFA production described in Chapter 3.1 provide VFA every day. Regarding the pairing of both productions steps (VFA and PHA production) and having in mind that with a cycle time of one day the highest PHA concentration (28.4% CDW) was yielded, a CT = 24 h is favourable [42].

### 3.3. Potential Analysis

#### 3.3.1. Calculation for German Waste Water Treatment Plants

**Figure 8:**
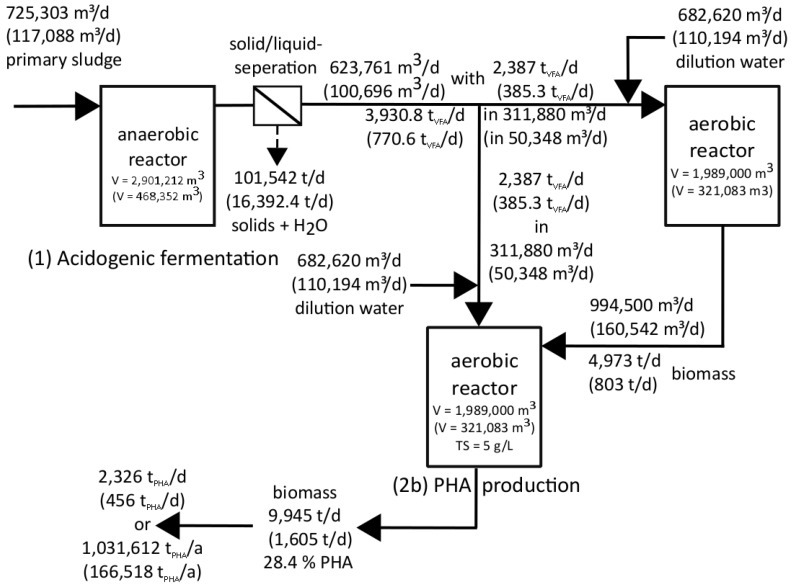
Biopolymer potential scheme for European and German (figures in brackets) WWTPs.

Figure 8 shows the results for possible biopolymer production on German WWTPs. All material streams and reactor volumes in this figure are theoretical values showing the size of the flows, if the best substrate for acidification [34] of all German WWTPs with preliminary sedimentation potential (PSP = more than 10,000 PE) would be used for PHA production.

First of all, the calculation for the amount of primary sludge in Germany regarding the PEs (Table 1) connected to German WWTPs is shown in Equation (6).

(6) $$115,700,000\;\mathrm{PE}\times 1.1\;\frac{L_{\mathrm{PS}}}{\mathrm{PE}\times d}\;=1,272,700,000\;\frac{L_{\mathrm{PS}}}{d}\;=127,270\;\frac{{m^{3}}_{\mathrm{PS}}}{d}$$

Around 92% of PEs are coming from WWTPs with preliminary sedimentation potential (Table 1), on which a primary clarifier is installed or the construction of a primary clarifier would be preferable. Thus, the actual amount of primary sludge is calculated as follows:

(7) $$127,270\;\frac{{m^{3}}_{\mathrm{PS}}}{d}\times 92\%=117,088.4\;\frac{{m^{3}}_{\mathrm{PS}}}{d}$$

Using these data and the results of own experiments [34,42] and of Chapter 3.1 and 3.2 a calculation of the possible PHA production through various steps can be performed.

Implementing the best reactor operation method, using a retention time of 4 days and a daily withdrawal of 25% (Table 1) 117,088.4 $\frac{m^{3}}{d}$ acidified material could be used for PHA production every day (Equation (8)).

(8) $$117,088.4\;\frac{{m^{3}}_{\mathrm{PS}}}{d}\times 4\;d\times 25\;\frac{\%}{d}=117,088.4\;\frac{m^{3}}{d}$$

The total solid (TS) concentration of the acidified material of 35 $\frac{\mathrm{kg}}{m^{3}}$ (Table 1) and the assumed residual moisture after de-watering via centrifuge of 75% leads to a daily acidified liquid production of 100,696 $\frac{m^{3}}{d}$ (Equations (9)–(11)).

(9) $$117,088.4\;\frac{m^{3}}{d}\times 35\;\frac{{\mathrm{kg}}_{\mathrm{TS}}}{m^{3}}=4,098,094\;\frac{{\mathrm{kg}}_{\mathrm{TS}}}{d}=4098.1\;\frac{t_{\mathrm{TS}}}{d}$$

The assumed residual moisture of 75% means that the calculated 4098.1 $\frac{t_{\mathrm{TS}}}{d}$ biomass is 25% of the total mass separated by the centrifuge. Accordingly, 75% of the separated total mass is water. Assuming that the solid phase is completely separated it follows:

(10) $$4098.1\;\frac{t_{\mathrm{TS}}}{d}+\frac{4098.1}{25}\frac{\frac{t_{\mathrm{TS}}}{d}}{\%}\times 75\%\;\frac{t_{H_{2}O}}{d}=16,392.4\;\frac{t_{\mathrm{TS}+H_{2}O}}{d}$$

With an assumed average density of 1 $\frac{t}{m^{3}}$ the amount of

(11) $$117,088.4\;\frac{m^{3}}{d}-\frac{16,392.4}{1}\;\frac{\frac{t}{d}}{\frac{t}{m^{3}}}=100,696\;\frac{m^{3}}{d}$$

of acidified liquid is available for the PHA production step.

Regarding the average VFA concentration of 7653 $\frac{{\mathrm{mg}}_{\mathrm{VFA}}}{L}$ (Table 1) the amount of VFA in the acidified water can be calculated (Equation (12)).

(12) $$7653\;\frac{g_{\mathrm{VFA}}}{m^{3}}\times 100,696\;\frac{m^{3}}{d}=770.6\;\frac{t_{\mathrm{VFA}}}{d}$$

The substrate is divided into equal parts to both reactors (2a and 2b) of the second production step so that each reactor is supplied with 50,348 $\frac{m^{3}}{d}$ of acidified liquid (Equation (13)), containing 385.3 $\frac{t_{\mathrm{VFA}}}{d}$ (Equation (14)).

(13) $$\frac{100,696\frac{m^{3}}{d}}{2}=50,348\;\frac{m^{3}}{d}$$

(14) $$\frac{770.6\;\frac{t_{\mathrm{VFA}}}{d}}{2}=385.3\;\frac{t_{\mathrm{VFA}}}{d}$$

In order to achieve the required loading rate of 1.2 $\frac{{\mathrm{kg}}_{\mathrm{VFA}}}{m^{3}\times d}$ in both reactors of the second production step, the volume of Reactor 2a (sum over Germany) and 2b (sum over Germany) should be 321,083 m^3^ each (Equation (15)).

(15) $$\frac{385,300\;\frac{{\mathrm{kg}}_{\mathrm{VFA}}}{d}}{1.2\;\frac{{\mathrm{kg}}_{\mathrm{VFA}}}{m^{3}\times d}}=321,083.3\;m^{3}$$

Reactor 2a is operated with a retention time of 2 days, a daily withdrawal of 50% of the reactors volume and a total solid concentration of TS = 5 $\frac{g_{\mathrm{TS}}}{L}$ (Table 1). Due to the fact that there is no biomass sedimentation before the removal of the material, the withdrawn material can be considered as fully mixed. Therefore, the amount of bacteria must double every cycle to achieve a constant concentration of total solids. During the own experiments it was shown that this necessary condition was fulfilled [42].

The removal of 50% of the reactor volume leads to the amount of substrate, which has to be filled in the Reactor 2b every day (Equation (16)).

(16) $$321,083.3\;\frac{m^{3}}{d}\times 50\%=160,541.7\;\frac{m^{3}}{d}$$

The emptying and refilling process at the beginning of every cycle is represented in Figure 3. In order to reach a VFA concentration of 1.2 $\frac{{\mathrm{kg}}_{\mathrm{VFA}}}{m^{3}\;\times \;d}$ and regarding to the fact that the VFA concentration at the end of a cycle is zero, a substrate with a VFA concentration of 2.4 $\frac{{\mathrm{kg}}_{\mathrm{VFA}}}{m^{3}\;\times \;d}$ is needed if half of the reactors volume is exchanged.

Equation (16) shows the required amount of substrate for one day for one reactor regarding the needed VFA concentration. Hence the amount of VFA rich liquid of 50,348 $\frac{m^{3}}{d}$ (Equation (13)) has to be diluted with 110,193.7 $\frac{m^{3}}{d}$ fresh water (Equation (17)).

(17) $$160,541.7\;\frac{m^{3}}{d}-50,348\;\frac{m^{3}}{d}=110,193.7\;\frac{m^{3}}{d}$$

When calculating the amount of dilution water it should be noted that the large amount of water is due to the reactors comparatively low total solid concentration of TS = 5 $\frac{g_{\mathrm{TS}}}{L}$, which was installed during the experiments in [42]. This concentration is used for the potential analysis as well, to keep the calculation as close as possible to the operation conditions of the carried out experiments. Of course, a much higher solid concentration could be installed, leading to significantly smaller reactor volumes as well as less dilution water. As the amount of dilution water does not affect the result of the potential analysis it was kept.

As described, the biomass concentration in Reactor 2a was 5 $\frac{g_{\mathrm{TS}}}{L}$ at the end of a cycle. Regarding the withdrawal of 50% of the reactor’s volume a total of

(18) $$321,083.3\;\frac{m^{3}}{d}\times 5\;\frac{{\mathrm{kg}}_{\mathrm{TS}}}{m^{3}}\times 5\%=802,708.3\;\frac{{\mathrm{kg}}_{\mathrm{TS}}}{d}=802.71\;\frac{t_{\mathrm{TS}}}{d}$$

biomass is transferred into Reactor 2b. This reactor also had a dry matter content of 5 $\frac{{\mathrm{kg}}_{\mathrm{TS}}}{m^{3}}$ after the PHA production step (Table 1). Considering a cycle time of one day and the volume of 321,083 m^3^, the amount of biomass in Reactor 2b sums up to (Equation (19)):

(19) $$\frac{321,083.3\;m^{3}}{1\;d}\;\times \;5\;\frac{{\mathrm{kg}}_{\mathrm{TS}}}{m^{3}}=1,605,416.5\;\frac{{\mathrm{kg}}_{\mathrm{TS}}}{d}\;=1605.4\;\frac{t_{\mathrm{TS}}}{d}$$

With the reached PHA concentration of 28.4% of the cell dry weight (CDW) [42] the daily amount of biopolymer is calculated in Equation (20).

(20) $$1605.4\;\frac{t_{\mathrm{TS}}}{d}\times 28.4\%\;\frac{\mathrm{PHA}}{\mathrm{TS}}=455.9\;\frac{t_{\mathrm{PHA}}}{d}$$

Finally, the possible annual amount of PHA production on German waste water treatment plants can be calculated in Equation (21).

(21) $$455.9\;\frac{t_{\mathrm{PHA}}}{d}\times 365.25\;\frac{d}{a}\;=166,517.5\;\frac{t_{\mathrm{PHA}}}{a}$$

Dividing the reactor volume (sum of all production stages) of 1,110,518 m^3^ by the people’s equivalent with preliminary sedimentation potential of 106.56 Mio. PE (Table 1) results in a reactor volume (sum of all production stages) per capita of around 10.4 L/PE. Each PE can contribute to the production of 1.6 kg_PHA_/(PE×a). Keeping in mind that the aeration tank volume on a WWTP with 100,000 PE sums up to 10,000 m^3^–15,000 m^3^ an additional reactor volume of approximately 1050 m^3^ would be needed for the biopolymer production, only. Thus, the extra volume would not be disproportional.

#### 3.3.2. Estimation for European Waste Water Treatment Plants

The Biopolymer potential for European WWTPs are calculated similar to Section 3.3.1 and also shown in Figure 8.

As there are missing data about the amount of connected persons (PEs) for many EU member states as well as about the amount of municipal waste water, it is impossible to calculate the EU-wide production of primary sludge analogous to Equations (6) and (7). However, there are data for all 28 EU member states regarding the production of sewage sludge. These data show the dry weight of sewage sludge in $\frac{t_{\mathrm{TS}}}{a}$ (Table 1) and hence their unit must be transferred into $\frac{m^{3}}{a}$ (Equation (22)) to compare them with the data used for German WWTP in Equation (6). Therefore, an average total solid concentration of 15 $\frac{g_{\mathrm{TS}}}{L}$ or 66.67 $\frac{m^{3}}{t_{\mathrm{TS}}}$ for European sewage sludge (primary and secondary) is assumed.

(22) $$13,245,180\;\frac{t_{\mathrm{TS}}}{a}\times 66.67\;\frac{m^{3}}{t_{\mathrm{TS}}}=883,056,150\;\frac{m^{3}}{a}$$

On the assumption that the proportion of primary sludge in the amount of sewage sludge is more or less constant in all member states, the percentage can be calculated (Equation (25)) using the theoretical yearly amount of primary sludge produced in Germany (Equations (6) and (23)) and the yearly amount of German sewage sludge (Table 1) (Equation (24)).

(23) $$115,700,000\;\mathrm{PE}\times 1.1\;\frac{L_{\mathrm{PS}}}{\mathrm{PE}\times d}\times 365.25\;\frac{d}{a}=46,485,367.5\;\frac{{m^{3}}_{\mathrm{PS}}}{a}$$

(24) $$1,815,150\;\frac{t_{\mathrm{TS}}}{a}\times 66.67\;\frac{m^{3}}{t_{\mathrm{TS}}}=121,016,051\;\frac{m^{3}}{a}$$

(25) $$\frac{46,485,367.5\;\frac{{m^{3}}_{\mathrm{PS}}}{a}}{121,016,051\;\frac{m^{3}}{a}}\times 100\%=38.4\%$$

Assuming that not all European waste water treatment plants are equipped with a primary clarifier, the proportion of primary sludge is rounded off to 30%, so that the yearly amount of European primary sludge sums up to 265 Mio. $\frac{{m^{3}}_{\mathrm{PS}}}{a}$ (Equation (26)) or 725,303 $\frac{{m^{3}}_{\mathrm{PS}}}{d}$ (Equation (27)).

(26) $$883,056,151\;\frac{{m^{3}}_{\mathrm{PS}}}{a}\times 30\%=264,916,845.3\;\frac{{m^{3}}_{\mathrm{PS}}}{a}$$

(27) $$\frac{264,916,845.3\;\frac{{m^{3}}_{\mathrm{PS}}}{a}}{365.25\;\frac{d}{a}}=725,302.8\;\frac{{m^{3}}_{\mathrm{PS}}}{d}$$

By now, the European biopolymer potential can be calculated analogous to Equations (8)–(21).

The amount of acidified material is:

(28) $$725,303\;\frac{{m^{3}}_{\mathrm{PS}}}{d}\times 4\;d\times 25\;\frac{\%}{d}=725,303\;\frac{m^{3}}{d}$$

Using Equations (9)–(11) the amount of acidified liquid can be calculated (Equation (31)):

(29) $$725,303\;\frac{m^{3}}{d}\times 35\;\frac{{\mathrm{kg}}_{\mathrm{TS}}}{m^{3}}=25,385,605\;\frac{{\mathrm{kg}}_{\mathrm{TS}}}{d}=25,385.6\;\frac{t_{\mathrm{TS}}}{d}$$

(30) $$25,385.6\;\frac{t_{\mathrm{TS}}}{d}+\frac{25,385.6\;\frac{t_{\mathrm{TS}}}{d}}{25\%}\times 75\%\;\frac{t_{H_{2}O}}{d}=101,542.4\;\frac{t_{\mathrm{TS}+H2O}}{d}$$

With an average density of 1 $\frac{t}{m^{3}}$

(31) $$725,303\;\frac{m^{3}}{d}-\frac{101,542.4\;\frac{t}{d}}{1\;\frac{t}{m^{3}}}=623,760.6\;\frac{m^{3}}{d}$$

of acidified liquid can be used within the second PHA production step. This leads to the amount of VFAs in the acidified water (Equation (32)):

(32) $$7653\;\frac{g_{\mathrm{VFA}}}{m^{3}}\times 623,760.6\;\frac{m^{3}}{d}=4773.6\;\frac{t_{\mathrm{VFA}}}{d}$$

Analogous to Equations (13) and (14) both reactors of the second production step are supplied with 311,880 $\frac{m^{3}}{d}$ (Equation (33)) of acidified liquid containing 2386.8 $\frac{t_{\mathrm{VFA}}}{d}$ (Equation (34)).

(33) $$\frac{623,760.6\;\frac{m^{3}}{d}}{2}=311,880.3\;\frac{m^{3}}{d}$$

(34) $$\frac{4773.6\;\frac{t_{\mathrm{VFA}}}{d}}{2}=2386.8\;\frac{t_{\mathrm{VFA}}}{d}$$

The reactor volumes (Equation (35)) can be calculated analogous to Equation (15):

(35) $$\frac{2,386,800\;\frac{{\mathrm{kg}}_{\mathrm{VFA}}}{d}}{1.2\;\frac{{\mathrm{kg}}_{\mathrm{VFA}}}{m^{3}\;\times \;d}}=1,989,000\;m^{3}$$

The daily substrate amount for one reactor is (Equation (36)):

(36) $$1,989,000\;\frac{m^{3}}{d}\times 50\%=994,500\;\frac{m^{3}}{d}$$

Analogous to Equation (17) the amount of dilution water can be calculated (Equation (37)):

(37) $$994,500\;\frac{m^{3}}{d}-311,880.3\;\frac{m^{3}}{d}=682,619.7\;\frac{m^{3}}{d}$$

With a withdrawal of 50% a biomass transfer to Reactor 2b of 4094.58 $\frac{t_{\mathrm{TS}}}{d}$ is necessary (Equation (38)).

(38) $$1,989,000\;\frac{m^{3}}{d}\times 5\;\frac{{\mathrm{kg}}_{\mathrm{TS}}}{m^{3}}\times 50\%=4,972,500\;\frac{{\mathrm{kg}}_{\mathrm{TS}}}{d}=4972.5\;\frac{t_{\mathrm{TS}}}{d}$$

With the described total solid concentration of 5 $\frac{{\mathrm{kg}}_{\mathrm{TS}}}{m^{3}}$ after the PHA production the amount of biomass in Reactor 2b is (Equation (39)):

(39) $$1,989,000\;m^{3}\times 5\frac{{\mathrm{kg}}_{\mathrm{TS}}}{m^{3}}=9,945,000\;\frac{{\mathrm{kg}}_{\mathrm{TS}}}{d}=9945\;\frac{t_{\mathrm{TS}}}{d}$$

With a cycle time of one day and the reached PHA concentration of 28.4% CDW (Table 1) the daily amount of PHA sums up to (Equation (40)):

(40) $$9945\;\frac{t_{\mathrm{TS}}}{d}\times 28.4\%\frac{\mathrm{PHA}}{\mathrm{TS}}=2824.4\;\frac{t_{\mathrm{PHA}}}{d}$$

Finally, the possible annual amount of PHA production on European waste water treatment plants can be calculated in Equation (41).

(41) $$2824.4\;\frac{t_{\mathrm{PHA}}}{d}\times 365.25\;\frac{d}{a}\;=1,031,612.1\;\frac{t_{\mathrm{PHA}}}{a}$$

#### 3.3.3. Summary of the Results and Optimization Potential

The market for biopolymers is predicted to grow continuously [43]. In 2016, a worldwide biopolymer production of 4.16 Mio.$\frac{t}{a}$, of which 861,120 $\frac{t}{a}$ or 20.7% [43] suit the criteria of the stringent definition for biopolymers, introduced in Chapter 1, were achieved. Taking Equation (21) into account approximately 4.0% of the worldwide biopolymer production (bio- and non-biodegradable) could be produced just by using primary sludge from German WWTPs. Around 19.3% of the worldwide biopolymers could be produced on WWTPs in Germany considering the stringent definition, only.

For the biopolymer production on European WWTPs (Equation (41)) approximately 24.7% of 2016’s worldwide biopolymer production (bio- and non-biodegradable) or around 119.8% of 2016’s worldwide biopolymer production due to the stringent definition could be produced.

Assuming an improved PHA production with an achievable PHA concentration of 0.5 $\frac{g_{\mathrm{PHA}}}{g_{\mathrm{VSS}}}$ [44] or even around 60% CDW [45,46] a total amount of 2,179,446.8 $\frac{t_{\mathrm{PHA}}}{a}$ (Equation (42)) could be produced on European WWTPs by using primary sludge, only.

(42) $$9945\;\frac{t_{\mathrm{TS}}}{d}\times 60\%\times 36,525\;\frac{d}{a}=2,179,446.8\;\frac{t_{\mathrm{PHA}}}{a}$$

Thus, approximately 52.4% of 2016’s worldwide biopolymer production (bio- and non-biodegradable) or 253.1% of 2016’s worldwide biopolymer production due to the stringent definition could be produced in an improved production on WWTPs in the EU.

A large proportion of polymers (biopolymers and those from synthetic production) is used for packing materials. The PHAs feature similar characteristics like polypropylene (PP), which is the mostly sold plastic in the EU with 18.8% (around 8.6 Mio. $\frac{t}{a}$ ) market share in 2012 [47]. The potential analysis for Germany equates to approximately 1.9% of the EUs PP production. Using the calculation for the EU for PHA production from primary sludge around 12.0% of the conventional PP sold in the EU could be substituted which is a significant potential.

#### 3.3.4. Plausibility Analysis

As some input parameters do have a strong effect to the calculations, a plausibility analysis was carried out. All critical parameters, like total solid concentration of the primary sludge, the daily amount of primary sludge per PE, or the daily amount of primary sludge per PE were analysed and considered plausible. A more detailed description of the plausibility analysis can be found in [28].

## 4. Summary and Conclusions

From the results, it could be concluded that the production of high amounts of VFAs with a stable VFA composition on a WWTP is possible. Using different raw materials shows a strong influences on degree of acidification and VFA composition. The VFA production and composition is strongly influenced by a pH-level change in the reactor. A semi-continuous operation method of the reactor with a short RT and small WD is preferable. With primary sludge as raw material no biomass recirculation is needed during the fermentation process.

The results showed that the produces VFA are suitable for PHA production in a second stage. This amount of PHA produced is strongly influenced by the reactors operating conditions (temperature, pH-level and substrate concentration), while the PHA composition is influenced by cycle time changes. At preferred conditions, a stable PHB/PHV composition was reached and both PHAs were produced in nearly the same proportion.

Nevertheless, further research is needed to couple both processes for constant and long term PHA production and for upscaling.

The results of the presented potential analysis clearly indicate the possibility to produce large amounts of PHAs on German and European WWTPs. It has been shown that municipal WWTPs could be used as a significant source for biopolymers and waste water is an important substituent for plant-based raw materials in the PHA production.

More than twice the amount of 2016's worldwide biopolymer production could be produced on European WWTPs with an upgraded operation. Thus, the production of biopolymers on waste water treatment plants contribute to a recycling of the organic material contained in waste water.

## Figures and Tables

**Table 1:** Input data used during the potential analysis.

Parameter	Unit	Value	Literature
Connected people equivalents (PE) on German WWTPs	Mio. PE	115.7	[29]
Proportion of PSP *-PEs regarding total PEs in Germany	%	92	[29]
PEs with PSP * in Germany	Mio. PE	106.56	
Amount of primary sludge per PE	L/(PE×d)	1.1	[36]
Total solid conc. of primary sludge/acidified material	g/L	35	[4]
VFA concentration	g_VFA_/m^3^	7,653	[4]
Retention time and withdrawal at the first production step	d; %/d	4; 25	[4]
Total solid concentration in the aerobic Reactors 2a/2b	g/L	5.0	[4]
Loading rate for PHA production	kg_VFA_/m^3^	1.2	[4]
Retention time and withdrawal at Reactor 2a	d and %/d	2 and 50	[4]
PHA proportion based on cell dry weight	CDW.‑%	28.4	[36]
Yearly sewage sludge amount in the EU	t_TS_/a	13,245,180	[30]
Yearly sewage sludge amount in Germany	t_TS_/a	1,815,150	[30]

* PSP = German WWTPs with preliminary sedimentation potential (PSP = more than 10,000 PE).

**Table 2:** Degree of acidification and VFA composition in dependence of substrate and operation conditions (batch-tests, 4 L).

Carbon Source	pH	Temperature	Max. Conc.	DA	Ac/Pro/Bu
		(°C)	(Day)	(%)	(%)
Primary sludge	6 *	30	9	31	52/48/0
Primary sludge	6 *	20	7	14	56/44/0
Primary sludge	4.6	30	10	14	41/59/0
Primary-/digested sludge	7	20	14	14	79/21/0
Primary sludge	4.5	20	15	13	42/58/0
Primary-/digested sludge	7.5	30	14	12	84/16/0
Excess sludge	7	30	5	10	59/20/20
Excess sludge	6.5	20	4	8	60/20/20
Primary-/digested sludge	6 *	30	5	7	75/25/0
Excess sludge	6 *	20	7	6	24/76/7
Excess sludge	6 *	30	5	6	67/33/0
Primary-/digested sludge	6 *	20	2	3	57/43/0
Excess-/digested sludge	6 *	30	4	3	100/0/0
Excess-/digested sludge	8	30	3	3	76/0/24
Excess-/digested sludge	7.5	20	7	2	100/0/0
Excess-/digested sludge	6 *	20	2	1	100/0/0

* Marks conditions pH-controlled.

**Table 3:** VFA composition and DA in dependence of pH or RT and WD.

Batch			Semi Continuous			
pH	Ac/Pro/Bu	DA	pH	RT and WD	Ac/Pro/Bu	DA
	(%)	(%)		(day and %)	(%)	(%)
6	45/51/4	29	6	4 and 25	49/38/13	15
6.5	37/61/2	29	6	4 and 50	46/47/7	14
7	28/72/0	39	6	4 and 75	48/45/7	14
8	60/37/3	29	6	6 and 25	51/38/11	22
10	45/31/8*	14	6	6 and 50	46/48/6	17
			6	8 and 25	46/41/13	20
			6	8 and 50	43/49/8	19

* Missing to 100% is formate.

**Table 4:** PHA production in dependence of two tested substrate concentrations.

Substrate Conc.	Temp.	pH	PHA	PHB/PHV
(mg_VFA_/L)	(°C)		(% CDW)	(% CDW/% CDW)
1200	20	*	13.2	7.0/6.2
1200	20	7	25.9	13.2/12.7
1200	30	*	3.4	2.3/1.1
2000	20	*	4.8	3.3/1.5
2000	20	7	1.8	<2/1.8
2000	30	*	5.8	3.8/2.0

* Marks conditions without pH-control.

**Table 5:** PHA production in dependence of the reactor temperature.

Temp.	pH	CT	PHA	PHB/PHV
(°C)		(h)	(% CDW)	(% CDW/% CDW)
15	7	24/48	4.2	2.5/1.7
15	8	24/48	3.9	2.5/1.4
20	7	24	25.9	13.2/12.7
20	*	24	13.2	7.0/6.2
30	7	24	0.6	<2/0.6
30	*	24	3.4	2.3/1.1

* Marks conditions without pH-control.

**Table 6:** PHA production in dependence of the pH.

pH	PHA	PHB/PHV
	(% CDW)	(% CDW/% CDW)
unc. (av. 9)	13.2	7.0/6.2
6	−	−
7	25.9	13.2/12.7
8	28.4	14.7/13.7
9	4.4	3.0/1.4

**Table 7:** PHA production in dependence of the cycle time.

CT	Feast/Famine	Feast/Famine-Ratio	PHA	PHB/PHV
(h)	(min)	(%/%)	(% CDW)	(% CDW/% CDW)
24	524/916	36/64	28.4	14.7/13.7
48	500/2380	17/83	18.3	8.0/10.3
72	448/3872	10/90	21.4	8.3/13.1
